# Understanding the Burden of Agriculturally Significant Vector-Borne and Parasitic Diseases in Kansas

**DOI:** 10.1089/vbz.2025.0023

**Published:** 2025-06-30

**Authors:** Kristin Michel, Nicole M. Ioerger, Ashlie M. Ake, Susan M. Hettenbach, Cassandra Olds, Dustin L. Pendell, James Stack, Stephen Higgs, Dana L. Vanlandingham

**Affiliations:** ^1^ Division of Biology, College of Arts and Sciences, Kansas State University, Manhattan, Kansas, USA.; ^2^ Department of Diagnostic Medicine/Pathobiology, College of Veterinary Medicine, Kansas State University, Manhattan, Kansas, USA.; ^3^ Biosecurity Research Institute, Kansas State University, Manhattan, Kansas, USA.; ^4^ Department of Entomology, College of Agriculture, Kansas State University, Manhattan, Kansas, USA.; ^5^ Department of Agricultural Economics, College of Agriculture, Kansas State University, Manhattan, Kansas, USA.; ^6^ Department of Plant Pathology, College of Agriculture, Kansas State University, Manhattan, Kansas, USA.

**Keywords:** livestock diseases, one health, parasitic diseases, plant diseases, tick-borne diseases, vector-borne diseases

## Abstract

**Background:**

The state of Kansas (KS) has been called the “agricultural heartland” of the United States. Vector-borne and parasitic diseases (VBPD) have a major impact on the production of livestock, such as cattle, swine, goats and sheep, as well as crops, such as wheat, corn, and sorghum. The purpose of this review is to educate agricultural professionals in the state of KS about VBPD of current or potential concern and to inform the public about the challenges faced by the agricultural community.

**Methods:**

This review describes and discusses the endemic VBPD that currently impact agricultural production in KS and foreign VBPD of concern. In addition, we outline the major arthropod vectors of VBPD in KS, including ticks, mites, and various insects. In the context of this review, parasites are strictly limited to arthropod ectoparasites that negatively impact livestock production. Modern agricultural data for the state of KS were mostly sourced from the USDA National Agricultural Statistics Service, and current KS VBPD data were mostly sourced from the KS State Veterinary Diagnostic Laboratory.

**Conclusion:**

These VBPD have a large economic impact on the state and country, and we have concluded there is a need for updated estimates regarding the economic burden of VBPD in KS and throughout the United States to make better animal and crop health investment decisions.

## Introduction

The state of Kansas (KS) is located at the center of the contiguous United States and has an area of 82,278 square miles. Approximately 90% of the state is used for agricultural purposes, making KS the “agricultural heartland” of the United States (Britannica, 2020). It has a temperate climate with periods of extreme heat and extreme cold and, in recent years, an agricultural growing season from mid-to-late February to mid-October (Sittel, 2024). As the state with the most total acres of cropland in the country, KS was the top producer of sorghum for grain and winter wheat in 2023 (NASS, 2024b). Agricultural animal production is also a major contributor to KS’s economy, with over $14 billion in sales in 2022 (Table 1) (NASS, 2024c).

**Table 1.** Agricultural Animal and Crop Market Value Data for the State of Kansas in 2022

	Total sales (USD)
Cattle and calves	13,596,819,000
Hogs and piglets	854,805,000
Sheep and goats	26,991,000
Corn	*3,002,965,000
Wheat	*1,509,375,000
Sorghum	*818,636,000
Total	19,809,591,000

* 2023. Source: USDA, National Agricultural Statistics Service 2024c.

Vector-borne and parasitic diseases (VBPD) pose significant challenges to animal, plant, and human health. Throughout the world, endemic and sporadic outbreaks and potential introductions of VBPD threaten agricultural and public health sectors. The purpose of this article is to educate individuals involved in agricultural occupations at all levels in KS about the VBPD with current or potential impact within the state and to inform the public about the challenges faced by our agricultural community. Specifically, this review summarizes the major VBPD that affect the agricultural sector within the state of KS, primarily focusing on pathogens transmitted by ticks, mites, and insects to cattle, swine, sheep, corn, wheat, and sorghum. Also included are selected exotic VBPDs that should be considered as posing a threat to the agricultural animals in KS because of the possibility of being introduced into the state. There are zoonotic VBPD transmitted by mosquitoes to birds in KS. However, the two main pathogens, West Nile virus (WNV) and St. Louis encephalitis virus, primarily affect wild bird populations and do not pose a direct threat to poultry-based agricultural production (Oyer et al., 2014); therefore, they are not included, but a recently published article reviews the history of human WNV infections in KS (Higgs et al., 2025). In addition, while the equine industry is important to agriculture in KS, VBPD of horses were not included because the paper’s scope was limited to food animals and crops.

VBPD can cause significant economic burden within the agricultural sector. Economic impacts from diseases in livestock stem mainly from reduction in marketable products, reduction in product quality, increased inputs (*i.e.,* additional feed needed to achieve the same rate of weight gain), costs related to disease prevention and control (*i.e.,* vaccines, treatments, eradication of diseased animals), costs to protect human health, animal welfare considerations (*i.e.,* reduction in animal suffering), and international trade restrictions imposed to control disease spread (Bennett, 2003; Bennett and Ijpelaar, 2005; McInerney, 1996). Additional economic losses may stem from damaged consumer perception of animal products following an outbreak (McInerney, 1996). VBPD of agricultural plants also cause economic losses due to slowed or stunted plant growth, diminished crop quality, decreased yields, and costs associated with disease prevention and treatment (Gai and Wang, 2024). Economists strive to create models that generate information regarding the real costs of diseases and areas of feasible change to reduce losses (Gilbert et al., 2024). Initiatives such as the Global Burden of Crop Loss, the Global Burden of Animal Diseases, and the World Health Organization for Animal Health’s Collaborating Center for Animal Health Economics—Americas Region, strive to improve methods for estimating the economic burden of globally important animal and plant diseases. However, gaps still exist (Gilbert et al., 2024; Pendell et al., 2024; Rushton et al., 2018; Szyniszewska et al., 2024), and estimates regarding the true economic burden for many of the VBPD of importance in KS are currently lacking. Better understanding regarding the cost-benefit dynamics of these diseases can lead to better animal and crop health investment decisions and more efficient usage of limited resources (Kappes et al., 2023).

## The Agricultural Sectors Affected by VBPD in KS

### Cattle

With over 6 million head of cattle and sales of over $13 billion (Table 1), KS was the second highest cattle producing state in the United States in 2022 (NASS, 2024a). VBPD transmitted by ticks, midges, and biting flies have a major impact on cattle production in KS, directly due to decreased production and indirectly due to transport and export restrictions that are imposed when animals become infected. One VBPD of particular importance in KS is the tick-vectored disease bovine anaplasmosis, which is caused by a rickettsial pathogen that infects red blood cells, leading to severe anemia and sometimes infertility, abortions, and sudden death (Kocan, 2010). One study found an overall anaplasmosis infection prevalence of 51% in KS cow-calf operations (Spare et al., 2020). Biting flies also pose a substantial economic and health burden to cattle, as well as other livestock species, in KS due to “fly worry,” or the reduction in growth and production caused by fly-induced pain and suffering (Byford et al., 1992; Campbell et al., 2001; Catangui et al., 1997; Price, 2017; Taylor et al., 2012; Wright, 1985). Two endemic viral diseases, bluetongue and epizootic hemorrhagic disease, both transmitted by *Culicoides* midges, are also relatively common pathogens in KS based on seroprevalence. However, they do not often cause serious disease in cattle (McVey et al., 2024). Vesicular stomatitis (VS) and bovine theileriosis are present in KS but only cause sporadic outbreaks. Rift Valley fever virus (RVFV) is not currently present but is of concern due to the anticipated economic impact of a potential introduction (Table 2).

**Table 2.** Vector-Borne and Parasitic Diseases of Cattle in Kansas (Agents with the Most Disease Burden and/or Economic Impact are Listed First)

Disease	Pathogen name	Pathogen type	General vector	Specific vector (s)	Status in cattle in KS
Bovine anaplasmosis	*Anaplasma marginale*	Bacteria	Ticks and biting flies	*Dermacentor variabilis* (American dog tick) and *Stomoxys calcitrans* (stable fly)	Present (Spare et al., 2020)
Fly worry (fly-induced pain and suffering)	NA	NA	Flies	*Stomoxys calcitrans* (stable fly), *Haematobia irritans irritans* (horn fly), *etc.*	Present (Swist et al., 2002, Trehal et al., 2017)
Bluetongue (BT)	Bluetongue virus (BTV)	Virus	*Culicoides* midges	*Culicoides sonorensis* (McVey et al., 2024)	Present (McVey et al., 2024)
Epizootic hemorrhagic disease (EHD)	Epizootic hemorrhagic disease virus (EHDV)	Virus	*Culicoides* midges	*Culicoides sonorensis* (McVey et al., 2024)	Present (McVey et al., 2024)
Vesicular stomatitis (VS)	Vesicular stomatitis Indiana virus (VSIV)	Virus	Biting insects	*Culicoides,* sand flies, and black flies (Pelzel-McCluskey et al., 2021)	Uncommon (Pelzel-McCluskey et al., 2021)
Bovine theileriosis	*Theileria orientalis*	Protozoan	Ticks	*Haemaphysalis longicornis* (Asian longhorned tick)	Uncommon (APHIS, 2024)
Rift valley fever (RVF)	Rift valley fever virus (RVFV)	Virus	Mosquitoes	Mainly *Aedes* and *Culex* spp. (Balaraman et al., 2024)	Not present—of concern (Balaraman et al., 2024)

### Swine

Currently, VS Indiana virus is uncommonly transmitted to swine in KS by biting midges, sand flies, and black flies (Pelzel-McCluskey et al., 2021). In addition, two viruses are of concern as potential introductions (Table 3). African swine fever virus (ASFV) is a tick-transmitted virus that, if introduced to the United States, could cause substantial economic losses to swine production. Although this virus is often transmitted through fomites and pig-to-pig contact, it can also be transmitted by soft ticks (Brown and Bevins, 2018; Groocock et al., 1980; Hess et al., 1987) and by stable flies when they take a blood meal (Mellor et al., 1987) or are ingested by susceptible pigs (Olesen et al., 2018). The mosquito-transmitted Japanese encephalitis virus (JEV) is also not currently present in the United States but is a virus of concern due to its potential to cause abortions and stillbirths, which would pose a substantial economic threat to the swine industry (Oliveira et al., 2019). Recent studies have demonstrated the competence of common North American species of mosquitoes as vectors of JEV (Auerswald et al., 2021; Huang et al., 2015) and the susceptibility of both domestic and feral phenotype swine to infection with JEV (Park et al., 2018a; Park et al., 2023). Other studies have demonstrated the potential role of North American birds to be amplifying hosts for JEV (Nemeth et al., 2012). Since there is no justification for pre-emptive vaccination of the U.S. human population to protect them from JEV infection, most people would be highly susceptible if JEV were to be introduced into the United States.

**Table 3.** VBPD of Swine in Kansas (Agents with the Most Disease Burden and/or Economic Impact are Listed First)

Disease	Pathogen name	Pathogen type	General vector	Specific vector	Status in swine in KS
Fly worry (fly-induced pain and suffering)	NA	NA	Flies	*Stomoxys calcitrans* (stable fly), *Haematobia irritans irritans* (horn fly), *etc.*	Present (Swist et al., 2002, Trehal et al., 2017)
Vesicular stomatitis (VS)	Vesicular stomatitis Indiana virus (VSIV)	Virus	Biting insects	*Culicoides,* sand flies, and black flies (Pelzel-McCluskey et al., 2021)	Uncommon (Pelzel-McCluskey et al., 2021)
African swine fever (ASF)	African swine fever virus (ASFV)	Virus	Ticks and stable flies	*Ornithodoros* spp. (Brown and Bevins, 2018) and *Stomoxys calcitrans* (Mellor et al., 1987)	Not present—of concern (Brown and Bevins, 2018)
Japanese encephalitis (JE)	Japanese encephalitis virus (JEV)	Virus	Mosquitoes	Many species of *Culex, Culiseta, Ochlerotatus, Aedes, Anopheles, and Mansonia* have been identified as potential vectors (Oliveira et al., 2019)	Not present—of concern (Oliveira et al., 2019)

VBPD, vector-borne and parasitic diseases.

### Sheep and goats

Although the number of sheep and goats in the state of KS is smaller than cattle or swine, the economic benefit from the sheep and goat industry is still notable, with combined sales reaching over $26 million in 2022 (Table 1). VBPD that currently impact sheep and goat production in the region include bluetongue virus (BTV), spread by *Culicoides* biting midges, and Cache Valley virus (CVV), spread by mosquitoes (Table 4). Sheep and goats also uncommonly contract VS (Pelzel-McCluskey et al., 2021). Although RVFV is not currently found in the United States, it would pose a threat to sheep and goat agriculture if introduced due to the high rate of abortions it causes (Daubney et al., 1931; Oymans et al., 2020).

**Table 4.** VBPD of Sheep and Goats in Kansas (Agents with the Most Disease Burden and/or Economic Impact Are Listed First)

Disease	Pathogen name	Pathogen type	General vector	Specific vector	Status in sheep and goats in KS
Bluetongue (BT)	Bluetongue virus (BTV)	Virus	*Culicoides* midges	*Culicoides sonorensis* (McVey et al., 2024)	Present (McVey et al., 2024)
Fly worry (fly-induced pain and suffering)	NA	NA	Flies	*Stomoxys calcitrans* (stable fly), *Haematobia irritans irritans* (horn fly), *etc.*	Present (Swist et al., 2002, Trehal et al., 2017)
Vesicular stomatitis (VS)	Vesicular stomatitis Indiana virus (VSIV)	Virus	Biting insects	*Culicoides,* sand flies, and black flies (Pelzel-McCluskey et al., 2021)	Uncommon (Pelzel-McCluskey et al., 2021)
Cache Valley (CV)	Cache Valley virus (CVV)	Virus	Mosquitoes	At least 30 different mosquito species are potential vectors (Ayers et al., 2019)	Uncommon (Cino, 2020)
Rift Valley fever (RVF)	Rift Valley fever virus (RVFV)	Virus	Mosquitoes	Mainly *Aedes* and *Culex* spp. (Balaraman et al., 2024)	Not present—of concern (Balaraman et al., 2024)

VBPD, vector-borne and parasitic diseases.

### Wheat, corn, and sorghum

KS was the top wheat-producing state (NASS, 2024b) with over $1.5 billion in production value in 2023 (Table 1). Wheat streak mosaic (WSM) is the most important viral disease of wheat in KS, and it affects corn and sorghum plants as well (Table 5). Overlapping crop seasons provide a continuous green bridge, which supports the migration of wheat curl mites between plant systems and the spread of the WSM complex viruses (De Wolf et al., 2017). Barley yellow dwarf (BYD) is another important viral disease of cereals, including wheat, in KS. BYD was responsible for approximately 33% of annual wheat losses in KS between 2005 and 2013 (Enders et al., 2018). In addition to wheat, corn production is a highly profitable industry in KS with over $3 billion in production value in 2023 (Table 1). Maize chlorotic mottle virus (MCMV), in concert with any of several potyviruses (*i.e.,* maize dwarf mosaic virus, sugarcane mosaic virus, Johnson grass mosaic virus, or WSM virus), causes maize lethal necrosis (MLN) disease in corn plants. Vectored by thrips and originally described in the United States in KS, MCMV has spread across the U.S. corn belt and three other continents, where it is now causing major crop losses (Biswal et al., 2022; Redinbaugh and Stewart, 2018). Although, MLN has not caused significant losses in KS in recent years, it could re-emerge as a production constraint (Stewart, 2022). The potyviruses are also capable of infecting and causing mosaic disease in sorghum plants (Seifers and Kofoid, 1998). In addition, the corn leaf hopper, *Dalbulus maidis (D.* maidis), and the corn stunt pathogen, *Spiroplasma kunkelii*, were confirmed in KS for the first time in 2024. So far, corn stunt disease incidence is low, but it has been detected in 26 of 105 KS counties (Onofre et al., 2024). Corn stunt disease has been associated with corn crop losses of up to 70% (Pinto et al., 2024), so it may have a significant impact on corn production in KS.

**Table 5.** VBPD of Wheat, Corn, and Sorghum in Kansas

Disease	Pathogen name	Pathogen type	General vector	Specific vector	Status in wheat, corn, and sorghum in KS
Barley yellow dwarf (BYD)	Barley yellow dwarf virus (BYDV), Cereal yellow dwarf virus (CYDV), and Maize yellow dwarf virus (MYDV)	Virus	Aphids	Several aphid species, including *Rhopalosiphum padi, Sitobian avenue,* and *Schizaphis gramium* (De Wolf, 2018)	Present in wheat (Enders et al., 2018)
Corn stunt	*Spiroplasma kunkelii*	Mollicute	Corn Leafhopper	*Dalbulus maidis* (Onofre et al., 2024)	Present in corn (Onofre et al., 2024)
Maize lethal necrosis	Maize chlorotic mottle virus (MCMV) plus a potyvirus	Virus	Thrip	*Frankliniella occidentalis* (Redinbaugh and Stewart, 2018)	Present in corn (Stewart, 2022)
Mosaic disease	Sugarcane mosaic virus (SCMV), Sorghum Mosaic Virus (SrMV), Maize dwarf mosaic virus (MDMV), Johnsongrass mosaic virus (JGMV)	Virus	Aphids	Several aphid species, including *Rhopalosiphum maidis* and *Schizaphis gramium* (Seifers et al., 2012)	Present in corn and sorghum (Klein and Smith, 2020, Seifers and Kofoid, 1998)
Wheat streak mosaic (WSM)	Wheat streak mosaic virus (WSMV), High plains wheat mosaic virus (HPWMoV), and Triticum mosaic virus (TriMV)	Virus	Wheat curl mite	*Aceria tosichella* (Nachappa et al., 2021)	Present in wheat (Nachappa et al., 2021)

VBPD, vector-borne and parasitic diseases.

## Overview of Agriculturally Significant Pathogens and Their Vectors in KS

### Pathogens transmitted to livestock by ticks in KS

#### *Anaplasma marginale* (*A. marginale*), vectored by *dermacentor variabilis*

Bovine anaplasmosis, a disease caused by the bacterium *A. marginale*, was first described in KS in 1926 (Kocan et al., 2010). The disease is now widespread throughout the state (Spare et al., 2020), with 54 of 105 counties in KS reporting positive cases between the years of 2023 and 2024 (KSVDL, 2024). It can cause severe anemia, fever, icterus, lethargy, weight loss, abortions, and death in cattle, resulting in significant economic losses to the beef and dairy industries. Fatalities are most common in cattle over 2 years of age (Aubry and Geale, 2011), and those that survive the acute phase of infection become persistently infected (Kocan et al., 2010). The overall expected cost of anaplasmosis in cattle has been estimated at $660 per head (Railey and Marsh, 2021). The largest costs are related to treatment of infected animals and death loss, so monitoring *A. marginale* prevalence within a herd or region with a commercially available enzyme-linked immunosorbent assay can help minimize outbreaks and associated economic losses (Railey and Marsh, 2021).

*A. marginale* can be transmitted by ticks, biting flies, and fomites contaminated with infected blood. Approximately 20 species of ticks are thought to be capable vectors of *A. marginale* globally (Kocan et al., 2004), but the tick vector of most concern in KS is *Dermacentor variabilis*, also known as the American dog tick. It is one of the most common tick species in KS, with peak activity occurring in spring and summer (Hroobi et al., 2021, Ng’eno et al., 2024). Male *Dermacentor* ticks are known to become persistently infected with *A. marginale*, making them capable of repeatedly transmitting the disease agent when they transfer between cattle (Kocan et al., 2010). In addition to bovine anaplasmosis, *D. variabilis* also transmits the bacteria that cause tularemia and Rocky Mountain spotted fever. An increase in abundance of *D. variabilis* and shifts in its range possibly due to climate change may partially explain why these diseases have increased in incidence (Boorgula et al., 2020). Several studies have demonstrated that prescribed burning of pastures may offer an affordable, chemical-free method of decreasing tick burden on cattle (Gleim et al., 2019; Salazar et al., 2024; Victoria et al., 2013).

#### The threat of *theileria orientalis* (*T. orientalis*) via the spread of *haemaphysalis longicornis* (*H. longicornis*)

*T. orientalis* is a tick-vectored protozoan parasite capable of infecting red and white blood cells in cattle, resulting in infectious anemia. There are 11 genotypes of *T. orientalis*, but most do not cause clinical disease. The Ikeda and Chitose genotypes are pathogenic, causing anemia, abortion, fever, weakness, and death in infected cattle (Gebrekidan et al., 2020). Unlike *A. marginale*, *T. orientalis* is known to cause disease in calves under 2 years of age. After recovery, animals remain persistently infected reservoirs, and symptoms can reoccur in times of stress (Hanzlicek, 2022). To date, theileriosis outbreaks have been reported in 15 states in the United States, including KS (APHIS, 2024). The economic burden of bovine theileriosis in beef cattle involves losses due to quarantine, decreased production, and euthanasia of infected animals (Almazán et al., 2022), but overall estimates of economic losses in the United States are lacking.

*H. longicornis*, the Asian longhorned tick, is the primary tick vector of *T. orientalis* (Dinkel et al., 2021). It is an exotic tick species that was first detected in the United States in 2017 and has since been reported in 19 states (Ponnusamy et al., 2024). The spread of *H. longicornis* in the United States is facilitated over long distances by companion animal transport and over shorter distances by roaming wildlife (Egizi et al., 2020). Based on the latest data released by the USDA in February of 2023, *H. longicornis* was detected in Missouri, 50 miles east of the border with KS (APHIS, 2023). This species was also found on cattle in Craig County, Oklahoma, which borders KS to the south, in July of 2024 (Myers and Scimeca, 2024). It is an aggressive biter with a broad host range (humans, domestic animals, and wildlife), building intense infestations upon domestic hosts and causing great stress, reduced growth, and severe blood loss. Females can reproduce parthenogenetically, enabling the establishment of new populations through the introduction of a single female (Herrin and Oliver, 1974). A recent model predicts the spread of *H. longicornis* within the United States, with highly suitable habitat existing on the east and west coast and in several midwestern and southern states (Rochlin, 2019). If *H. longicornis* spreads into KS, it is feasible that cases of bovine theileriosis will become more common, threatening the state’s cattle industry. Currently, the dominant control strategy is the use of acaricides to control tick vector populations, but it is financially costly and the widespread use of these chemicals has resulted in increased tick resistance. Selectively breeding cattle that are genetically resistant to the disease may be a cost-effective alternative with promise for the future (Valente et al., 2022).

#### The threat of african swine fever virus, vectored by *ornithodoros* spp

ASFV is a large DNA virus and the only member of the family *Asfarviridae* and genus *Asfivirus*. ASFV is also unique because it is the only known DNA arbovirus. It infects wild and domestic swine, including African warthogs and giant forest hogs, which normally have inapparent infections, and European wild boars, which often show clinical signs. Clinical signs in domestic swine infected with highly virulent ASFV can be peracute with sudden death and a mortality rate near 100% after 6–20 days. Clinical signs with less virulent ASFV can be subacute or chronic (Fernández and White, 2016). This virus has been introduced, eradicated, and reintroduced into various countries in Europe and the Caribbean and, over the past 10 years, has been introduced into the Caucasus, Russia, and Asia. It is not currently found in the United States but is of concern as a potential introduction due to its recent expansion within Europe, Asia, Haiti, and the Dominican Republic (Brown and Bevins, 2018; Cochran et al., 2023; Ruiz-Saenz et al., 2022).

ASFV is highly stable in feed and soil and is transmitted through direct contact with infected pigs or fomites, consumption of contaminated food products, and tick bites (Cochran et al., 2023). Tick vectors include several species within the *Ornithodoros* genus (Hess et al., 1987). At least one of those species, *Ornithodoros turicata*, is present in KS. These ticks feed indiscriminately on numerous host species, including cattle and pigs, but can survive for years without feeding. They are long-lived and have highly adaptable feeding behaviors, which makes them excellent vectors (Donaldson et al., 2016). Considering KS is home to a known vector and host species of domestic and feral swine, it is possible that ASFV could become established in KS if it were to be introduced into the United States. The economic burden of such an outbreak would be significant considering there is no commercially available vaccine for ASFV in the United States and control relies heavily on culling all pigs within specified control zones (Cochran et al., 2023). In 2021, a publication from Iowa State University estimated the cost, were ASFV to enter the United States, would be anywhere from $15 billion to $50 billion (Carriquiry et al., 2021).

### Pathogens transmitted to livestock by mosquitoes in KS

#### CVV

CVV is a mosquito-borne orthobunyavirus that was first isolated from a *Culiseta inornata* mosquito in 1956 in Cache Valley, Utah, USA (Holden and Hess, 1959). The virus is now endemic in North America and has been isolated from at least 30 species of mosquitoes, but the primary vector species are unknown (Ayers et al., 2019). Infected mosquitoes can transmit the virus to cattle, sheep, goats, deer, and humans (Uehlinger et al., 2018). Based on serological studies, white-tailed deer are thought to be the main amplifying host (Blackmore and Grimstad, 1998; Muller et al., 2024). However, CVV is primarily a concern in pregnant ewes, because it causes embryonic death, abortion, and fetal malformations (Ayers et al., 2023; Edwards et al., 1989). In rare circumstances, CVV has also caused severe disease in humans even though humans are not part of the virus cycle and are considered incidental hosts (Ayers et al., 2023; Campbell et al., 2006; Wilson et al., 2017).

KS is home to *Anopheles punctipennis* and *Anopheles quadrimaculatus* (Garrison, 2018), which are both competent mosquito vectors of CVV (Andreadis et al., 2014). *Aedes* spp. found in KS are also competent vectors (Ayers et al., 2019). Cases of CVV in small ruminants are reported sporadically in KS, with positive cases being confirmed by the KS State Veterinary Diagnostic Laboratory as recently as 2020 (Cino, 2020). A 2011 study reported a 28% seroprevalence in sheep across 22 states, providing evidence that CVV infections are common throughout the United States (Meyers et al., 2015). There are currently no approved vaccines available for CVV (Ayers et al., 2023), although experimental vaccines have been evaluated and shown to induce an adequate neutralizing antibody response (Ayers et al., 2022; Dunlop et al., 2018). Due to inconsistency in the timing of outbreaks and the lack of longitudinal data in the United States, there are still considerable gaps in knowledge regarding appropriate control measures and the economic burden of CVV (Hughes et al., 2023).

#### The threat of RVFV

RVFV is a mosquito-borne phlebovirus that affects humans and livestock (Wang et al., 2022). It was first identified in Kenya in 1930 (Daubney et al., 1931), and is now endemic in the eastern and southern regions of Africa (Favier et al., 2006). It has caused major outbreaks in at least 11 countries in Africa and the Arabian Peninsula (Bird et al., 2009) and has spread geographically due to climate change, making it a hazard to nonepidemic countries (Bett et al., 2017; Fouque and Reeder, 2019; Iwamura et al., 2020; Kamal et al., 2018; Samy et al., 2016). Economically, sheep, goats, and cattle are the most important domestic animal hosts (Favier et al., 2006). RVFV-infected pregnant goats, sheep, and cattle experience high rates of spontaneous abortions and high fatality rates in young animals (Coetzer, 1982; Ikegami and Makino, 2011). Experimentally, RVFV can infect white-tailed deer, so the virus has the potential to also become established in wildlife if introduced to the United States (Gaudreault et al., 2015; Kakani et al., 2010; Wilson et al., 2018). The possible impacts of RVFV on North American agriculture and public health are considerable (Bird et al., 2009; Hartley et al., 2011; Pendell et al., 2016). Economic impacts on the agricultural industry could result from decreased production, culling of infected animals, decline in consumer demand for meat and dairy products, and international trade restrictions (Hartley et al., 2011; Pendell et al., 2016). In 2016, a model was created that predicted the entry of RVFV into KS via just 10 mosquitoes could cost the United States roughly $3.5 billion (Pendell et al., 2016). Due to its potential to be used as a bioweapon and absence of FDA-approved antiviral therapies or licensed vaccines for humans, RVFV is listed as a select agent by the Centers for Disease Control and Prevention and U.S. Department of Agriculture and classified as a Category A pathogen by the National Institutes of Health (Ayers et al., 2023a).

RVFV can be transmitted by numerous species of mosquitoes in several genera, including several species within the *Aedes* genus (Linthicum et al., 2016). KS is home to several *Aedes* species, including *Ae. vexans*, *Ae. albopictus*, and *Ae. aegypti* (Garrison, 2018), which have all been shown to be competent vectors for RVFV in a laboratory setting (Hoch et al., 1985; Turell et al., 1988; Turell et al., 2013). *Aedes* mosquitoes are floodwater species, which lay their eggs just above the waterline in aquatic habitats. Their eggs remain dormant during periods of drought and hatch when subsequently flooded with water. Field observations suggest that *Aedes* spp. are able to vertically transmit RVFV via transovarial transmission. The infected eggs remain dormant during dry spells only to hatch after a heavy rain (Linthicum et al., 1985). Therefore, *Aedes* spp. could play a key role in the maintenance of RVFV during winter and interepidemic periods if it were introduced into KS. In addition to *Aedes* spp., other mosquitoes in the *Culex* and *Anopheles* genus have been documented as important in the amplification of RVFV during outbreaks (Himeidan et al., 2014). In KS, *Culex tarsalis* may play a role as an important vector (Gorris et al., 2021; Turell et al., 2010). Other than mosquitoes, the virus infection is acquired through exposure to infectious aerosols, handling of aborted fetal tissue, or during slaughtering or necropsy of viremic animals (Meegan, 1981; Meegan et al., 1981; Van Velden et al., 1977). Control strategies for RVFV in the United States would likely involve the widespread use of insecticides targeting adult female mosquitoes alone or in combination with those that target mosquito larvae (Hartley et al., 2011).

#### The threat of JEV

JEV is a mosquito-borne flavivirus endemic to the Asia-Pacific region. Mosquitoes transmit the virus to Ardeid birds (herons, egrets, and bitterns), which act mostly as maintenance hosts, and pigs, which act as amplifying hosts. Humans and horses are dead-end hosts (Mackenzie et al., 2022). JEV is of significant agricultural concern to the United States, because it causes reproductive disease in pigs leading to abortions and stillbirths (Oliveira et al., 2019). The introduction of JEV into Australia in 2021 and its subsequent establishment (Williams et al., 2022) is evidence of the virus’s capacity to spread into new areas where all components of the transmission cycle are present, including susceptible mosquitoes (Huang et al., 2015; VDH, 2025) and susceptible domestic and feral phenotype pigs (Lyons et al., 2018; Park et al., 2018b). If introduced into the United States, the mechanisms by which the virus could survive the winter are unknown but could include transovarial transmission (Dixon et al., 2024; Mourya et al., 1991; Rosen et al., 1980; Rosen et al., 1978; Soman and Mourya, 1985). Economic losses associated with such an introduction have been predicted to total between $306 and $612 million in a 1-year outbreak scenario (Cook et al., 2024).

Susceptible species of mosquitoes, birds, and swine are present in KS. Susceptible species of mosquitoes in KS include *Culex pipiens* and *Culex quinquefasciatus* (Gorris et al., 2021). Both species have been shown to be competent vectors for JEV (Auerswald et al., 2021). While there is an approved vaccine for humans, there are currently no approved veterinary vaccines for JEV in the United States. Therefore, until a vaccine could be approved, control mechanisms would likely involve attempting to control mosquito populations around pig production facilities and limiting the movement of pigs (Monath, 2023). *Culex* mosquitoes are also known vectors of several other disease-causing viruses within and outside of the United States, including eastern equine encephalitis virus, western equine encephalitis virus, Venezuelan equine encephalitis virus, Zika virus, St. Louis encephalitis virus, and WNV (Gorris et al., 2021). Changes to average temperatures and precipitation due to climate change will likely cause shifts in *Culex* spp. distributions in the coming years, opening up opportunities for these diseases to shift into new geographic areas (Gorris et al., 2021). Since WNV and JEV are closely related and share the same vectors, if JEV were to be introduced here it could likely become established just as WNV has been. However, differences in the transmission cycle, notably the importance of swine as an amplifying host for JEV but not WNV, mean that extrapolation of distribution and incidences must be made with caution.

### Pathogens transmitted to livestock by biting midges in KS

BTV and epizootic hemorrhagic disease virus (EHDV) are both in genus *Orbivirus*, and both are endemic in the United States (MacLachlan, 1994; McVey et al., 2024). One study examining samples collected in KS in 2016 found that seroprevalence rates of BTV and/or EHDV in cattle were 76–100% (McVey et al., 2024). BTV primarily infects sheep and cattle, while EHDV primarily infects deer and cattle. The symptoms of the two diseases are clinically indistinguishable and can include hemorrhaging, edema, cyanosis, inflammation of the coronary bands at the base of the hooves, reproductive issues, and death. Due to the similarity in symptoms, cases of either virus are often collectively referred to as hemorrhagic disease (Mullen and Murphree, 2019). Morbidity and mortality associated with BTV infection are highly variable but generally much lower in cattle than in sheep (Saminathan et al., 2020). Although serologic studies suggest that EHDV infections are common throughout the United States, most cases result in no or mild clinical disease (Mullen and Murphree, 2019). BTV and EHDV management focuses on prevention, because there are no cures or treatments. Therefore, trade and movement restrictions are often exercised to prevent the spread of these diseases (Rivera et al., 2021).

Both BTV and EHDV are vectored by *Culicoides*, or biting midges, that feed on many species of animals and humans. In the United States and Canada, 151 species of *Culicoides* have been identified, but only two species*, C. sonorensis* and *C. insignis*, meet the criteria of a vector species established by the World Health Organization (McGregor et al., 2022). *C. sonorensis* is currently the only confirmed vector species of BTV and EHDV in KS, but *C. stellifer* may also be a vector (McGregor et al., 2022). Most cases of BTV and EHDV occur in the late summer or fall, which corresponds to the time of year when *C. sonorensis* hits peak abundance (Savini et al., 2011). When midges feed on animals infected with BTV, the virus moves to their salivary glands where it replicates over the next 10–20 days. At that point, midges can transmit the virus and will remain infected for the rest of their lifespan (Mullen and Murphree, 2019).

*Culicoides* spp., in addition to black flies and sand flies, are also suspected vectors of the causative agents of VS. VS is caused by two *Vesiculoviruses*: VS New Jersey virus and VS Indiana virus (VSIV). There are sporadic outbreaks of VS in the United States every 2–10 years, which can affect horses, cattle, swine, and other ruminant populations. KS was the state with the most VS cases caused by VSIV in 2020 with 196 affected premises in 26 counties. Clinical signs of VS include blister-like lesions, which form on various body surfaces. Lesions of the mouth can cause anorexia, while lesions of the coronary bands can result in lameness. Humans can also become infected through direct contact with the lesions (Pelzel-McCluskey et al., 2021). The symptoms of VS are similar to those associated with the highly virulent foot and mouth disease, so outbreaks of VS are generally treated with great concern. Economic losses result from trade and livestock movement disruptions, cancellation of livestock shows and auctions, decreased production, and culling (Hayek et al., 1998; Pelzel-McCluskey et al., 2021).

### Pathogens transmitted to plants by mites in KS

WSM virus complex is a family of three viruses that infect wheat plants, including WSM virus (WSMV), high plains wheat mosaic virus (HPWMoV), and triticum mosaic virus (TriMV). These viruses exist worldwide, and outbreaks can cause yield losses surpassing 50%. Yellow and green streaks appear on the leaves of infected plants, forming a mosaic pattern. Symptoms may then progress to leaf necrosis and plant death (Edde, 2022). WSMV is present and widespread in KS. A particularly bad outbreak in KS in 2017 resulted in the loss of 5.6% of wheat crop yields and $76.8 million in lost revenue (Nachappa et al., 2021). One study from 2010 estimated the economic impact of WSMV outbreaks at between $118.1/ha and $464.5/ha (Velandia et al., 2010).The current trend toward warmer global temperatures unfortunately has the potential to make these types of outbreaks more frequent and more severe (Fouque and Reeder, 2019).

The wheat curl mite, *Aceria tocichella*, is the vector for WSMV. It is a wormlike, soft-bodied mite that attacks wheat and various grass species. They feed on the whorls of developing leaves, causing leaf curling and reduction of the plant’s photosynthetic ability. The nymphs and adults are capable of transmitting several viruses to wheat plants and other cereal crops (Edde, 2022). There are two genetically distinct *A*. *tosichella* lineages that act as pests of wheat (Redila et al., 2021; Skoracka et al., 2018). Both biotypes, 1 and 2, are present in KS fields (Khalaf et al., 2020). Biotype 1 and 2 lineages differ in their ability to transmit WSMV, HPWMoV, and TriMV, with biotype 2 being the most efficient (Seifers et al., 2009). These mites are challenging to control, because they move via wind currents and survive on multiple plant hosts. Insecticides have been shown to be ineffective and cost prohibitive (Murphy and Burrows, 2021). More effective control strategies include removing volunteer wheat and grassy weeds at least two weeks before planting new wheat, avoiding planting too early when wheat curl mite populations are high, and choosing wheat varieties with natural resistance to WSMV (De Wolf et al., 2017).

### Pathogens transmitted to plants by aphids in KS

BYD is an aphid-vectored viral disease of global importance. BYD is caused by viruses in the *Luteoviridae* family, including BYD virus (BYDV), cereal yellow dwarf virus, and maize yellow dwarf virus (Walls et al., 2019). In KS, BYD has the most significant effect in the southeastern and central portions of the state. It can affect several species of grasses, including wheat, oat, and barley plants, causing stunted plant growth and yellow or red leaf discoloration. Leaves may also show black spots or streaking, and plant heads may have dark discoloration with shriveled grains. Yield losses depend on how developed the plants are at the time of infection. If plants are infected in autumn, it can result in yield losses greater than 35% (De Wolf, 2018).

It is estimated that aphids transmit nearly 30% of all plant viruses, making them the most important plant disease vectors (Véronique et al., 2010). Several species of aphids are known to vector BYD in KS, including *Rhopalosiphum padi (R. padi)*, *Sitobian avenue*, and *Schizaphis gramium* (De Wolf, 2018). *R. padi*, the bird cherry-oat aphid, is the species most associated with yield losses due to BYD in KS. Adults can be winged or wingless with winged individuals emerging under stressful conditions as a mechanism to travel in search of healthy plant hosts. They can reproduce asexually and have rapid development, allowing them to quickly achieve large population sizes. They generally inflict little physical damage on crops while feeding unless infestations are heavy, so most damage is caused by the viruses they transmit. The best management strategy for *R. padi* involves delaying planting of host plant species to reduce chances of infestations occurring in the fall season (Whitworth et al., 2022).

### Pathogens transmitted to plants by leafhoppers in KS

Corn stunt disease, which is caused by the bacterial pathogen *Spiroplasma kunkelii* and vectored by the corn leafhopper, is characterized by stunted growth, red or purple streaks, and chlorotic stripes on corn plant leaves. Infected corn plants may also produce many smaller ears of corn with missing kernels. Due to its high prevalence and association with yield losses up to 70%, corn stunt is considered to be the most significant disease of corn in the Americas (Pinto et al., 2024).

The corn leafhopper, *D. maidis*, and corn stunt disease have historically been limited to Texas, Florida, and California within the United States. However, *D. maidis* was reported in KS for the first time in 2024, shortly after being reported in Oklahoma and Missouri. Corn leafhoppers are easily identifiable due to two distinct spots found between their antennae and eyes, which is a unique feature of this species (Onofre et al., 2024). In addition to transmitting corn stunt spiroplasma (CSS), corn leafhoppers are also capable of vectoring maize bushy stunt phytoplasma and maize rayado fino virus (Pinto et al., 2024). So far, only CSS has been detected in KS, but surveillance for the other two pathogens is ongoing (Onofre et al., 2024). In addition to the indirect damage caused by pathogen transmission, corn leafhoppers directly damage corn crops by feeding on the plants’ sap and injecting toxins (Pinto et al., 2024). Cost-effective control methods for *D. maidis* and CSS are lacking. Efficacy for the use of insecticides has not been established in KS, and insecticides have the potential to negatively impact other beneficial insect species (Hruska and Peralta, 1997). However, one study has shown that applying neonicotinoid insecticides directly to seeds before planting can significantly decrease corn yield losses due to *D. maidis* infestations (Neves et al., 2022). It has not been determined whether the corn leafhopper or the spiroplasma will survive the winter in KS and, hence, require reintroduction in subsequent years; a vector monitoring system has been established (Onofre et al., 2024).

## Important Ectoparasites of Livestock in KS

In addition to vector-borne pathogen transmission, blood-feeding insects, such as the biting flies *Stomoxys calcitrans* (stable fly) and *Haematobia irritans irritans* (horn fly), are of critical concern to the livestock industry (Machtinger et al., 2021). Stable flies are temporary ectoparasites that contact an individual host animal only briefly (2–5 min) to feed once or twice a day and move between different host animals (Bishopp, 1913). Although they are poor vectors, they have been shown to be competent in transmitting disease-causing pathogens such as equine infectious anemia virus and *A. marginale* (Foil et al., 1983; Scoles et al., 2005). Stable fly populations can reach high levels, particularly in the spring and early summer such that their poor vectorial capacity may be overcome by sheer numbers. In addition, they cause mechanical damage with their painful bites, resulting in reduced production and an estimated economic impact of $2.66 billion annually in the United States (Taylor, 2022). Horn flies remain associated with their animal host for the duration of their adult life stage and can be mechanical vectors of *Staphylococcus aureus*, a causative agent of summer mastitis, which is an important disease in dairy farming (Bramley et al., 1985; Owens et al., 1998). The economic threshold to implement control measures for horn flies has been established at ≥200 flies per animal (Hogsette et al., 1991). However, the economic impact caused by horn flies varies by animal breed and production type, age, environmental stressors, and other contributing arthropods. Land use patterns may also affect parasitism. In Oklahoma, patch burning resulted in a 41% reduction in horn flies (Scasta et al., 2012). This suggests that annual pasture burning, which is a widespread practice in KS, may reduce the impact of horn fly parasitism.

## Discussion

It is important to understand the regional economic burden of VBPD of agricultural animals and plants to help determine how such diseases should be prioritized and investigated going forward. Reviewing the literature for VBPD of agricultural importance in KS revealed that economic estimates for many of the diseases are lacking. For example, estimates for the economic burden of bovine theileriosis, CVV, EHDV, and corn stunt in the United States could not be found. Estimates for other VBPD are significantly outdated. A commonly quoted estimate for the cost of BTV is $3 billion globally, but the original source of that estimate is an article from 1989 (Bath, 1989). Similarly, commonly quoted estimates for the economic impact of horn flies are $700 million to $1 billion in losses to the U.S. beef cattle industry and $60 million spent on insecticides annually, but those estimates were calculated in the 1990’s (Byford et al., 1992; Cupp et al., 1998; Kunz et al., 1991). Estimates that VS has an economic impact of $100 to $200 per head or a mean loss of $15,565 per ranch are still being cited even though those estimates were made in 1985 and 1998, respectively (Goodger et al., 1985; Hayek et al., 1998). Estimates of economic cost for agricultural plant diseases are also outdated. For example, the most recent estimate for BYDV in the United States stated that a 5% yield reduction due to BYDV in the United States would result in losses of $847 million for corn, $387.1 million for wheat, $48.5 million for barley, and $28 million for oats. However, that estimate was calculated in 1995, based on data from 1989 (Hewings and Eastman, 1995). In addition, most publications discussing the impacts of VBPD in crops only discuss losses in terms of reduction in crop yields (Byamukama et al., 2014; Zhao and Zhou, 2025), but very few translate those yield losses into dollar amounts.

It is clear that there is still much to discover or update regarding the costs associated with VBPD in agricultural animals and crops in the United States. While it is understandable that research is being conducted on foreign diseases that could enter the United States due to the impact those viruses would have on the economic landscape, it is equally important that we continue to investigate the impact of endemic diseases. Agriculture has changed dramatically in the United States since most of the existing economic impact studies were conducted. These changes include a movement away from small family-owned farms toward larger operations, changes in production technologies that allow for more animals to be tended by one person, and an increased specialization of farms (ERS, 2020; MacDonald and McBride, 2009). In addition, global climate change is expected to continue to impact weather trends and the frequency of extreme weather events as well as vector and agent distributions over time (Fouque and Reeder, 2019). Understanding of how such changes impact disease spread and their associated economic impacts will be vital to make better animal and crop health investments now and in the future.

Considering the financial burden that could be caused by a disease outbreak in KS, various agencies participate in research, surveillance, outbreak response preparedness, and the implementation of biosecurity measures for animal and plant diseases throughout the state. Kansas State University in Manhattan, KS is home to the Biosecurity Research Institute, which houses biosafety level 3 facilities for researching VBPD. The National Bio and Agro-defense Facility is also located in Manhattan and provides biosafety level 4 facilities for disease research. The Kansas State Veterinary Diagnostic Lab provides plant and animal disease diagnostic testing that allows for the surveillance and reporting of disease occurrences throughout the state and beyond. In addition, Kansas State University is home to the National Agricultural Biosecurity Center whose mission is to help with the prevention and response to emerging threats to agriculture and the food supply in the United States. These facilities and organizations and many others play a vital role in protecting the health and safety of the agricultural industry throughout the great state of KS and the United States.
